# The Biggest Challenge for Prediction of Membrane Permeability of Cyclic Peptides: Scarce Data Availability

**DOI:** 10.1021/acs.jmedchem.6c00507

**Published:** 2026-03-03

**Authors:** Christina Lamers

**Affiliations:** Institute for Drug Discovery, Leipzig University, Brüderstr. 34, 04103 Leipzig, Germany

## Abstract

AI-based multiparameter optimization for membrane permeability of cyclic peptides is hampered by the limited availability of ground truth biological data. This Viewpoint highlights the Featured Article reporting an AI model, integrating a high-throughput assay classifying billions of cyclic peptides for permeability-related characteristics. Combining this with a generative AI, the first de novo designed permeable cyclic peptides containing polar groups were reported.

Cyclic peptides have gained increasing interest in drug discovery due to their ability to bind challenging targets, such as protein–protein interfaces. They furthermore engage their targets with high affinity and selectivity, due to their high constraints of the macrocyclic structure, reducing the entropic penalty upon binding. Additionally, the cyclic structures render them more stable against proteases and can increase membrane permeability compared to linear peptides. Nevertheless, cell permeability remains a big challenge for cyclic peptides, hampering their development for intracellular targets and limiting their oral bioavailability.

The Featured Article by Baker et al. tackles the biggest hurdle for cyclic peptides: achieving membrane permeability while retaining sequences showing solubility and that resemble FDA-approved structures. This is a multimodal optimization problem, where AI-models can be of use if there is a big enough data set for training and prediction. Baker et al. introduce PEGASUS, a multimodal AI model, which includes an innovative assay to generate large amounts of ground truth data on permeability-related characteristics, with solvent-dependent computational simulations and a generative AI learning from these acquired data. The significance of this study lies in the assay, which enables the generation of a massive and unbiased data set of permeability-related characteristics of cyclic peptides with drug-relevant sequences. This will substantially bring forward the design and prediction of membrane-permeable cyclic peptides. Solving the challenge of membrane permeability for peptides is recognized as the holy grail in the field of peptide therapeutics, unleashing the full potential of peptide therapeutics toward an application for intracellular targets and oral administration.

Traditionally, peptides had been perceived as a difficult therapeutic modality due to their pharmacokinetic challenges of limited plasma stability, fast renal clearance, and their detrimental membrane permeability.^1^ Therefore, peptide therapeutics have been developed for parenteral application, which often comes with limited acceptance by the patients. That limited the use of peptides to severe chronic diseases (e.g., cancer or diabetes). In the 1980s, the most prominent example of a cell-permeable peptide therapeutic – cyclosporin, an immunosuppressant cyclic peptide natural product from *Tolypocladium inflatum* – reached the market and revolutionized transplantation medicine. Extensive research on this prime example of a membrane-permeable peptide has highlighted the role of H-bonds and overall lipophilicity. Cyclosporine features *N*-methylated amide bonds, reducing the polarity of the backbone. Importantly, the conformational flexibility of cyclosporine enables the formation of intramolecular H-bonds for the passage through the membrane, while exposing the H-bonds in an aqueous environment to confer solubility. This chameleonic behavior is one important characteristic of its membrane permeability. The features identified for cyclosporine have also been systematically investigated by the groups of Lokey,^2^ Fairlie, and Craik,^3^ synthesizing libraries of short, head-to-tail cyclized peptides with *N*-methylated and lipophilic amino acids (Figure 1). Early computational studies have utilized these data points to predict cell permeability. However, those studies are very limited in the covered chemical space of the peptides and the number of data points. A recently published data set on cyclic peptides with correlated permeability data (CycPeptMPDB)^4^ sparked a variety of more recent predictive machine-learning studies. Still, the underlying data set covers a limited chemical space of mainly aliphatic amino acids, not overlapping with the chemical space of FDA-approved peptides. A recent approach of generating experimental data on a novel peptide library was reported by Nielson et al, using a high-throughput synthetic method combined with a live cell assay to detect cell permeability. Nevertheless, this approach is also limited in the generation of data points to a bit more than hundred.^5^

**Figure 1:**
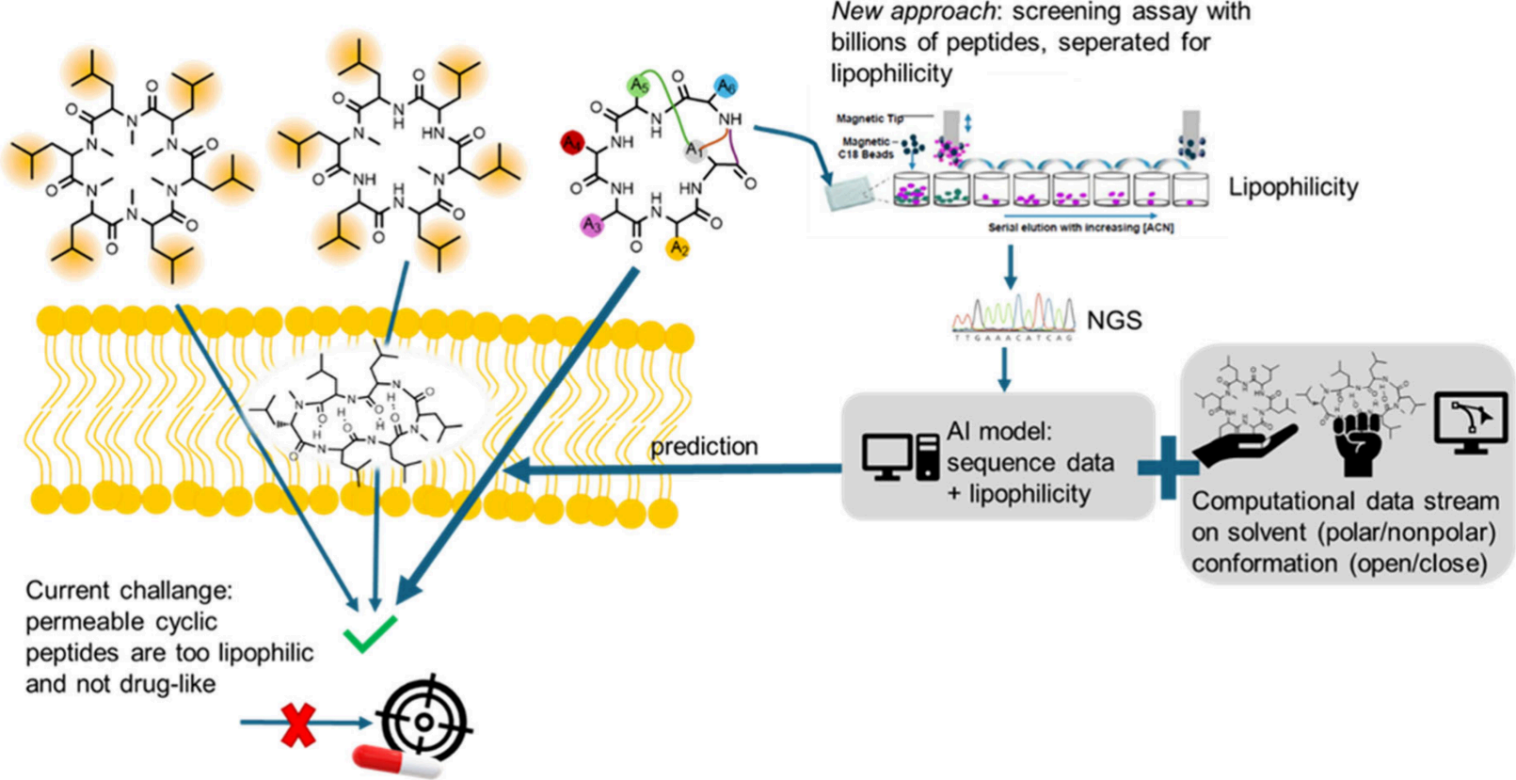
Much research has been conducted to identify features of cyclic peptides supporting membrane permeability; traditional approaches use head-to-tail cyclized peptides with lipophilic side chains (e.g., Leu) or *N*-methylated peptides, which tend to form intramolecular hydrogen-bonds upon membrane passage in a chameleonic behavior. However, these peptides often lack affinity to specific targets. The described PEGASUS pipeline from Baker et al. uses a high-throughput assay (“1910 PPA”) of mRNA-encoded peptides of all flavors to select for features of lipophilicity. The peptides with interesting lipophilic behavior are identified by next-generation-sequencing (NGS) and the data fed into an AI model. This is combined with a computational data stream predicting the conformation in polar and nonpolar solvent, to predict the open vs closed conformation, necessary for membrane passage. Hereby cyclic peptides with polar side chains are predicted as membrane permeable, enabling the design of relevant cyclic peptides of FDA-approved peptide features.

PEGASUS integrates a high-throughput assay (“1910 proxy permeability assay (PPA)”) to determine the lipophilicity of cyclic peptides, which is based on mRNA-display to generate an unbiased peptide library of 2.6 × 10^10^ different cyclic peptides (Figure 1). The usage of NNK codons enables the statistical integration of all canonical amino acids, thereby generating a diverse library, which is not limited to a certain set of amino acids, e.g., aliphatic ones. In mRNA display, the peptides stay connected to their encoding DNA, which enables the amplification of the signal by PCR and the identification of each peptide by sequencing. To fractionate the library into pools of peptides based on their hydrophobicity, C18-coated magnetic beads were used, from which the peptides were eluted into increasing concentrations of acetonitrile. To identify the peptide sequences in each fraction, the attached cDNA of each sample is PCR-amplified and submitted to NGS. It has been well established that the retention time of cyclic peptides on a chromatographic column (C18 is mostly used) correlates with their lipophilicity and polar surface area (PSA), which are relevant parameters for membrane permeability.^6^ However, the chromatic determination is time and solvent-intensive and not suitable for evaluating a library. Furthermore, the prediction of the lipophilicity and PSA of peptides is complex due to the conformational flexibility and the strong role of intramolecular H-bonds in peptides. This is especially true for cyclic peptides, where the cyclization can lead to a big change in chromatographic behavior.

For each peptide, the hydrophobicity was calculated based on established methods to verify if the separation into fractions correlated with hydrophobicity. In addition to the separation based on C18, PLRP-S beads were used. In total, 2.7 billion valid reads and 563 million unique peptide-fraction data points have been created and used to train the AI/ML models. The study is especially exciting, since the data set is not only limited to head-to-tail cyclic peptides, but also contains disulfide and thioether cyclized examples, which are cyclization methods often found in bioactive peptides. Complementary to the high-throughput assay, a physics-based computational workflow was developed in this study, using conformational sampling using meta-dynamics simulations, and solvent effects were modeled using an analytical linearized Poisson–Boltzmann model, enabling large-scale conformational ensemble generation. Summarizing all analyses, the study defines Permeability-optimized lipophilicity (POL) rules, including ChromlogD_7.4_ (threshold >2.2) and cLogP (threshold <4), balancing solubility and permeability-enhancing features. To obtain meaningful cyclic peptides, the authors analyzed the characteristics of FDA-approved peptides and condensed these into the generation of 33 de novo cyclic peptides, of which the majority showed high solubility. A total of 17 peptides had been tested in a cell-based permeability assay utilizing MDCK-MDR1 cells, out of which four showed permeability values high enough for oral availability.

The gain of interest in cyclic peptides for drug discovery was especially driven by technological advancements, which have led the field from peptide-hormone derivatives and peptides derived from natural sources into the era of de novo designed peptides. With pegcetacoplan and zilucoplan, the first approved cyclic peptides evolved by Phage display and mRNA-display, respectively, have reached the market, proving the success of screening random, huge libraries to identify binding peptides for any target. Furthermore, the advancement of structural biology is fueling the rational design of protein-domain-mimetics, such as α-helical PPI, while the development of AI-diffusion methods for cyclic peptides is under full speed. Those methods already enable the design of novel bioactive peptides; however, the parallel optimization toward membrane permeability is still a challenge. This is especially true, as the identified physicochemical features for membrane permeability are often in sharp contrast to the features that convey binding affinity, specificity, and solubility.

This especially underlines the beauty of the here reported approach; by using mRNA-Display to divide the peptide library into sublibraries based on features supporting membrane permeability, a favorable sublibrary could subsequently be submitted to the selection against the target of interest, while a sublibrary of target-binding peptides could be further selected for their lipophilic behavior: This enables the identification of peptides combining affinity to the target and features correlated with membrane permeability. However, it must be noted that the described assay focuses on features supporting passive diffusion, while membrane permeability can also be mediated by transporter- and receptor-mediated processes, limiting the predictiveness for membrane permeability, and even more for oral availability, where also proteolytic and pH stability are relevant.

In summary, the importance of this study lies in the acquisition of a huge data set on sequences of cyclic peptides annotated with their lipophilicity. As the quality of AI models is limited by the size and quality of the data set on which the models were trained, high-quality data will make AI-models more powerful and successful. One example of the importance of data availability is the great advancements of computational protein-structure prediction, which was only achievable due to the public availability of structural data in the PDB. The field of membrane permeability for peptides will only thrive to a similar extent if all data is publicly available, which is unfortunately not the case for the data generated here by the 1910 PPA assay.
